# Investigation of the Relationship Between Subjective Symptoms of Visual Fatigue and Visual Functions

**DOI:** 10.3389/fnins.2021.686740

**Published:** 2021-07-15

**Authors:** Fuhao Zheng, Fang Hou, Ruru Chen, Jianhui Mei, Pingping Huang, Bingzhen Chen, Yuwen Wang

**Affiliations:** Eye Hospital, Wenzhou Medical University, Wenzhou, China

**Keywords:** visual fatigue, contrast sensitivity, subjective symptoms, vergence facility, binocular accommodative facility, factor analysis, binocular vision

## Abstract

**Purpose:**

To investigate whether the severity of symptoms of visual fatigue might be associated with clinical visual measures and basic visual functions, such as accommodation, vergence, and contrast sensitivity.

**Methods:**

In this study, 104 students were recruited (25 males, 79 females, Age 23.4 ± 2.5) for this study. Those with high myopia, strabismus, anisometropia, eye disease or history of ophthalmological surgery were excluded. The included subjects completed a questionnaire that assesses the severity of visual fatigue. Then, binocular accommodative facility, vergence facility and contrast sensitivity using a quick contrast sensitivity function approach were measured in a random sequence. Next, the correlations between each symptom of visual fatigue in the questionnaire and accommodative facility, vergence facility and contrast sensitivity were examined.

**Results:**

Factor analysis indicated that visual fatigue, as captured by the scores of a subset of the questionnaire items, could be strongly related to binocular accommodative facility and binocular contrast sensitivity, but not to vergence facility. We also found that binocular accommodative facility and contrast sensitivity at high spatial frequencies are related.

**Conclusion:**

Our findings suggest that visual fatigue is related to the ability of human observers to encode visual details through their binocular vision.

## Introduction

Visual fatigue refers to a group of somatic or perceptive symptoms that usually occur following using a computer, reading, or other performing near visual activities (Bhanderi et al., 2008). The prevalence of visual fatigue is 12.4-32.2% in children below 18 years (Ip et al., 2006; Sterner et al., 2006; Tiwari et al., 2011; Tiwari, 2013) and 46–71% in university students around the world (Bhanderi et al., 2008; Han et al., 2013; Hashemi et al., 2019). Moreover, the prevalence of visual fatigue has been increasing (Sheppard and Wolffsohn, 2018).

Common symptoms of visual fatigue are blurred vision, diplopia, and illusory movement or flicker of words at a near viewing distance. These characteristics are related to near vision and binocular anomalies (Chen, 1986; Sheedy et al., 2003; Blehm et al., 2005; García-Muñoz et al., 2014). A questionnaire has been used as a quick method to assess the severity of symptoms and distinguish patients with symptoms from those who have normal vision (García-Muñoz et al., 2014).

Visual acuity is measured to evaluate the severity of visual fatigue. For instance, the larger the visual extent required to resolve a spatial pattern, the more severe the visual fatigue (Leroy, 2016). However, measurement of visual acuity might not be ideal. For example, blurred vision is a cardinal symptom of visual fatigue. Patients who suffer from visual fatigue often complain about blurred vision. However, studies show that their visual acuity is normal (Vilela et al., 2015). A 10-year follow-up study about visual fatigue reveals no relationship among visual fatigue and age, sex, seniority of work, visual acuity, and refractory disorders (Larese et al., 2019). In addition, a visual acuity test only utilizes optotypes with high degrees of contrast. Therefore, it might not reflect the visual performance in the real world (Marmor, 1986) where visual targets could appear at a relatively lower contrast, such as high spatial frequency content. High spatial frequency information can appear at a relatively lower contrast because the contrast sensitivity for higher spatial frequency content is much lower in humans. For this reason, measurement of contrast sensitivity across spatial frequency might better capture the ability of human observers to detect and encode the details (Arden, 1978; Marmor, 1986). In addition, studies show that contrast sensitivity, but not visual acuity, is impaired in patients who have visual disorders such as high myopia, asthenopia, foggy vision, and ocular hypertension (Quant, 1992; Järvinen and Hyvärinen, 1997; Gandolfi et al., 2005). These findings suggest that contrast sensitivity, rather than visual acuity, might be a more appropriate measure to diagnose a wide range of visual disorders.

Additionally, visual fatigue can occur to demands on early visual functions such as focusing and converging the eyes at a near object (Wilkins, 1995; Scheiman and Wick, 2014). Thus, accommodative and binocular dysfunctions might play a pivotal role in causing visual fatigue (Rosenfield, 2011; Sheppard and Wolffsohn, 2018; Golebiowski et al., 2020). Accommodation function is measured by accommodative amplitude and accommodative facility. Vergence function is measured by phoria, fusional range reserves, and vergence facility (Scheiman and Wick, 2014). Importantly, facility of accommodation and binocular vision can be more informative than the amplitude of accommodation and vergence (Liu et al., 1979; Hennessey et al., 1984; Scheiman and Wick, 2014) for clinical assessment. For instance, vergence facility and accommodative facility are central indexes of binocular vision (Buzzelli, 1991; Grisham et al., 2007; Palomo-Alvarez and Puell, 2008; Dusek et al., 2010; Quaid and Simpson, 2013; Scheiman and Wick, 2014). Many patients suffering from visual fatigue experience a decline in vergence or accommodative facility (Hennessey et al., 1984; Levine et al., 1985; Momeni-Moghaddam et al., 2014). Moreover, patients, whose amplitudes of accommodation and vergence are normal, have been shown to have severe visual symptoms if their accommodative and vergence facility are abnormal (Gall and Wick, 2003).

In this study, we investigated relationships between subjective symptoms (i.e., each item score of the questionnaire) and results from visual measurements, such as contrast sensitivity and accommodative and vergence facility. Contrast sensitivity, accommodation, vergence were measured using a qCSF method and lenses and prism flippers in 104 college students, respectively.

## Materials and Methods

### Participants

This study followed the tenets of the Declaration of Helsinki and was approved by the Hospital Committee of Wenzhou Medical University for the Protection of Human Subjects. For this study, 104 college students, 25 males and 79 females, ranged from 18 to 30 years (*M* = 23.4, *SD* = 2.5), were recruited from Wenzhou Medical University. Those with eye disease, ocular surgery, history of amblyopic or strabismus had been excluded. Their refractive error was between +1.00 Diopter (D) and −6.00 Diopter (D), astigmatism was less than 1.25 D. Their visual acuity was above or corrected to 20/20 with a normal monocular accommodative amplitude. All subjects were naïve to the purpose of the study.

### Questionnaire

A questionnaire of visual discomfort (Yang and James, 2011) was used to evaluate subjective symptoms of visual fatigue (see Appendix Table 1). In this paper, we define subjective symptom as each item in the questionnaire. Before their clinical examination, all subjects were asked to finish the questionnaire. Scores of the questionnaire for each symptom ranged from 0 (none) to 4 (extremely so). Visual fatigue was defined as the presence of one or more visual symptoms (subjective symptoms #2,3,6,7,9,10,13) (Han et al., 2013). In our study, there were about 53 of 104 (50.96%) participants who had more than one subjective symptom (score of symptoms is no less than two, which is the mean of the empirical distribution of the scores from all our participants) in the questionnaire, so they were categorized as having visual fatigue.

### Procedure

First, all subjects were asked to finish the questionnaire. Then accommodative facility and vergence facility were examined in a brightly lit room. Binocular contrast sensitivity was measured in a dark room on another day. All subjects performed the tests with best-corrected glasses. Some subject had good visual acuity without glasses, but some were corrected to 20/20. They had to wear their best-corrected glasses if they had ametropia.

### Accommodative Facility test

Binocular accommodative facility was measured using a flipper lens. A card had 6/9 (20/30) sized of letters. The card was positioned at a viewing distance of 40 cm. The participants were asked to report if the letters were clear while they were viewed with alternating +2.00 D and −2.00 D lenses. As an index of binocular accommodative facility, the number of cycles per minute (cpm) was measured. It denotes the ability of the observers to clear the plus lens followed by the minus lens. Any difficulties in responding between +2.00 D and −2.00 D was noted (Chen et al., 2021). F(+) means that the subjects had more difficulty in +2.00 D, whereas F(−) means that they experienced more difficulty in −2.00 D.

### Vergence Facility test

Vergence facility was evaluated at a viewing distance of 40 cm with a flipper prism. The power for the flippers was chosen as 3^△^BI/12^△^BO. A vertical column of small letter “E” at a 20/30 [6/9] size was presented as an accommodative target. The subjects observed the fixation target while their vision underwent best-correction. They reported when there was a clear and one visual target. We changed the flipper prism from BI to BO and from BO to BI, which makes up one cycle. Vergence facility was measured as cycles per minute, which is the number of cycles that each observer reported as clear and single visual percept for 1 min. In other words, this test measured the ability of the participants to form a fusion through BI and BO prisms. We noted if there were difficulty in the fusion through the BI and BO prisms during testing (Momeni-Moghaddam et al., 2014).

### Quick CSF test

We measured binocular contrast sensitivity using a qCSF method, which was written in MATLAB (MathWorks, Natick, MA) with PsychToolBox extensions (Kleiner et al., 2007). Stimuli (Zheng et al., 2018) were displayed on a gamma-corrected screen (KD-55 × 9300E, SONY, Tokyo, Japan). The display had a spatial resolution of 1920 × 1080 pixels and a refresh rate of 60 Hz. Each pixel subtended 0.908° at a viewing distance of 4 m. Observers viewed the display binocularly with their best corrections in a dark room.

**FIGURE 1:**
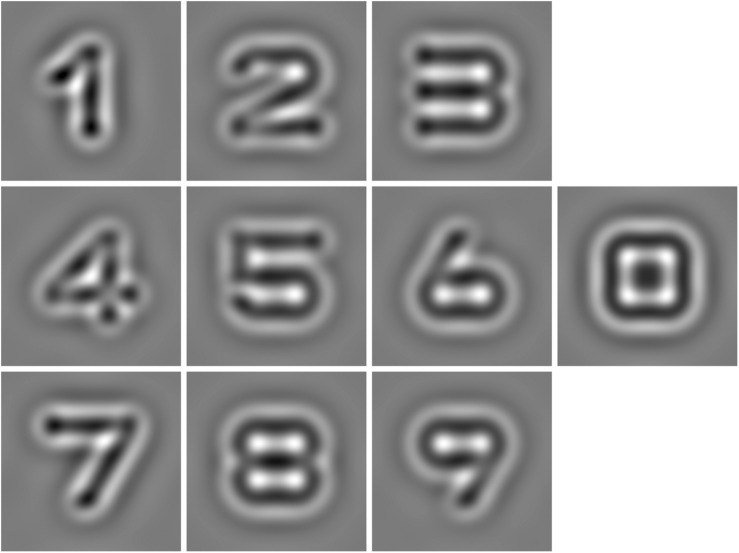
Ten Band-pass filtered digits were used as stimuli in the qCSF method.

In the CSF test, the participants were asked to perform a 10-digit identification task. Ten digits stimuli (0∼9, see Figure 1) were filtered using a raised cosine filter (Chung et al., 2002; Hou et al., 2016). The quick CSF changes the stimulus parameters based on the most recent response of the observer. In other words, a quick CSF algorithm automatically selects the most optimal contrast and spatial frequency of the stimulus for each subsequent trial (Lesmes et al., 2010). The responses of the observer revised posterior probability of the stimulus parameters.

Before starting the experiment, the observers spent 5 min adapting to the dark in the testing room. Besides the stimulus with the optimal contrast and spatial frequency (selected from the Bayesian posterior probability), two stimuli at higher contrasts were displayed during each trial. The presentation of these two additional stimuli enhanced the subject’s task performance. The three digits were randomly sampled from the 10 digits stimuli with replacement. Their positions were aligned in a single row; the distance between the neighboring digits was 1.1 times of the letter size. These three digits were displayed at the same spatial frequency but not in contrast.

Observers verbally identified the numbers. The experimenter recorded the response with the computer. When uncertain, observers reported by saying “I don’t know,” which was categorized as incorrect. There was no feedback to the response. A new trial began 500 ms after the subjects verbally identified the numbers (Hou et al., 2016).

### Data Analysis

The number of cpm of binocular accommodative facility and vergence facility was analyzed. There were 21 subjects who experienced difficulty during the accommodative facility test for +2.00D, and 7 subjects for −2.00D, two of whom had double vision in −2.00D. For vergence facility, there were 16 subjects who experienced difficulty in achieving fusion in 12^△^BO and 3 subjects in 3^△^BI. Since the number of subjects of infacility was too small to yield a reliable statistical result, we did not do further analysis.

**FIGURE 2:**
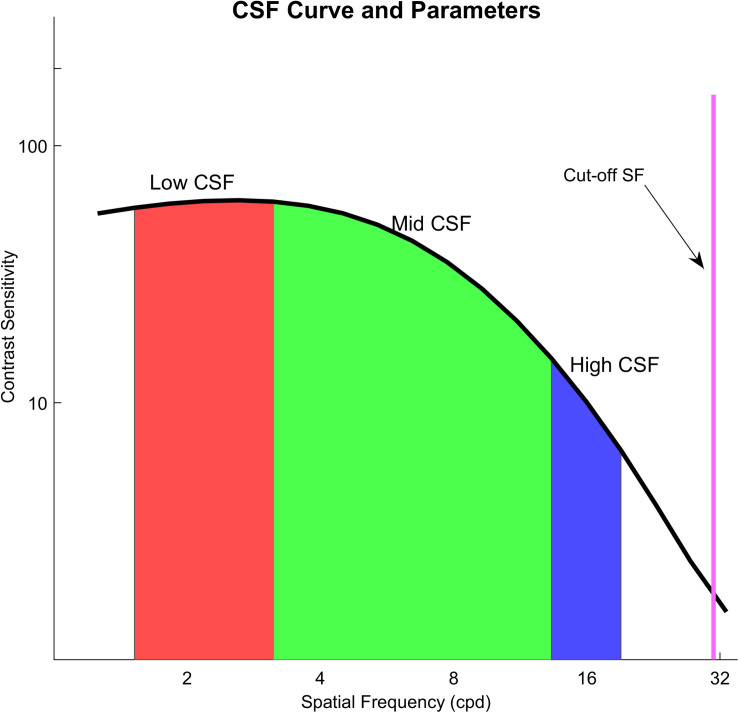
The black curve in the figure is the contrast sensitivity curve; the red area means an area under low frequency (1.5–3 cpd) of the CSF curve (Low CSF); the green area means the area under middle frequency (3–12 cpd) of the CSF curve (Mid CSF); the blue area means the area under high spatial frequency (12–18 cpd) of the CSF curve (High CSF); the purple line shows Cut-off SF.

We used a qCSF method, which is a parametric procedure, to estimate the entire CSF curve. After estimating a CSF, we computed the area under a curve. We used the areal measure as an index to describe contrast sensitivity as a function of spatial frequency. We computed four variations of the areal measure. These were an area under low frequency (1.5–3 cpd) of the CSF curve (Low CSF), the area under middle frequency (3–12 cpd) of the CSF curve (Mid CSF), the area under high spatial frequency (12–18 cpd) of the CSF curve (High CSF), and the area under the log CSF curve (AULCSF), which is a summary metric of the CSF function (Applegate et al., 1998; Oshika et al., 2006; Yan et al., 2010). They have been reported that the area under log CSF curve is correlated with optical aberration of the human eye and has been used as an image quality indicator (Barten, 1999). The area in different spatial frequency ranges may be a powerful metric to represent different aspects of visual performance. Moreover, a cutoff spatial frequency (Cut-off SF) was calculated; it is a spatial frequency at which the contrast threshold of CSF is 0.50 and characterizes the high frequency resolution of the visual system (Regan and Beverley, 1983; Kwon and Legge, 2011; Zhang et al., 2015). The illustration of these areal measures and Cut-off SF is shown in Figure 2.

A Shapiro-Wilk test was used to evaluate whether the data were normally distributed. The score of each item in the questionnaire, data of Low CSF and Cut-off SF had non-normal distributions. On the other hand, datasets of binocular accommodative facility, vergence facility, Mid CSF, High CSF had normal distributions. We compared binocular accommodative facility, vergence facility and contrast sensitivity between participants with or without visual fatigue. For normally distributed datasets, we used methods such as two independent sample *t*-test or a Pearson correlation test. Otherwise, we used a Mann-Whitney *U* test or a Spearman correlation test.

To map out the association between different subjective symptoms, we performed factor analysis using 15 items of the questionnaire. All the scores from the questionnaire were first standardized before factor analysis. To confirm whether factor analysis was appropriate for our datasets, we performed the Kaiser-Meyer-Olkin (KMO) test, which revealed 0.817; this is much higher than the necessary 0.7 which warrants factor analysis. Moreover, we performed Bartlett’s Test of Sphericity to see if the scores of the 15 items were unrelated to each other. It revealed *p* < 0.05, indicating that our datasets were related to each other. Hence, Bartlett’s Test also indicated that factor analysis was necessary.

Factor analysis enabled us to find latent factors (exogenous variables) that influenced the scores of the items in the questionnaire (endogenous variables). Next, we also examined the relationship between the latent factors and clinical measurements of visual functions, such as Cut-off SF, binocular accommodative facility, and vergence facility. Varimax with Kaiser Normalization was the rotation method to find rotated component. To make matrix clearer, we suppressed small coefficients when their absolute values were below 0.50. Then, multiple linear regression was used among variates with significant correlation.

The statistical analyses were performed using the software package SPSS (Windows version 22.0; IBM-SPSS). As for statistical significance, an alpha of 0.05 was established to reject the null hypothesis. When necessary, Bonferroni correction was applied.

## Results

**FIGURE 3:**
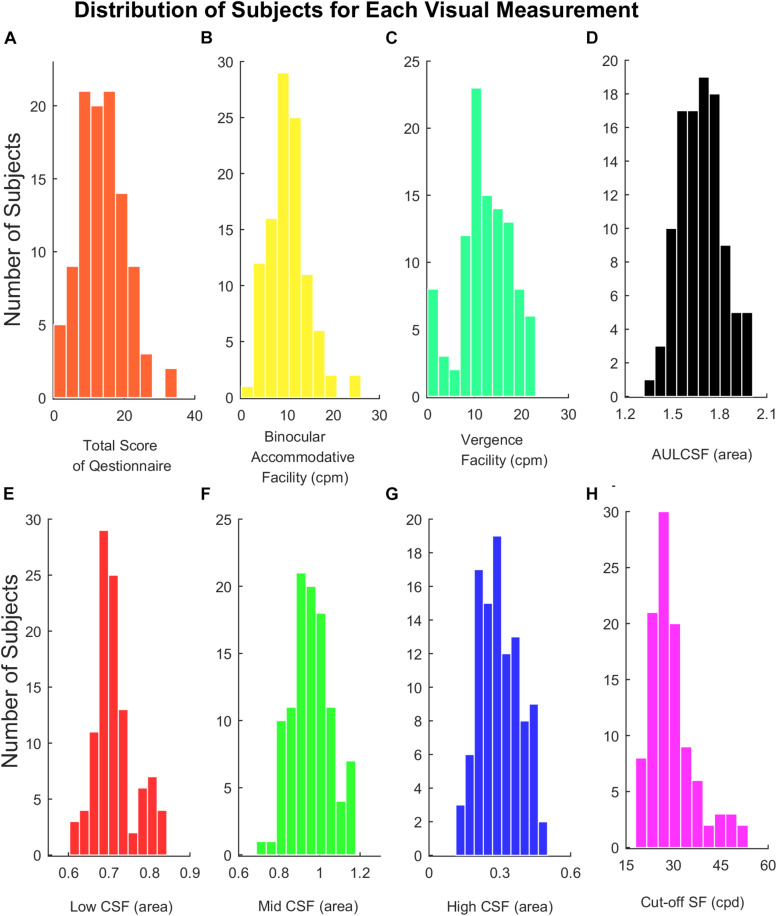
**(A)** Histograms of total score of the questionnaire. **(B)** Binocular accommodative facility. **(C)** Vergence facility. **(D)** AULCSF. **(E)** Low CSF. **(F)** Mid CSF. **(G)** High CSF. **(H)** Cut-off SF.

In our study, scores of the subjective symptoms in the questionnaire, visual acuity, Low CSF, Cut-off SF were not normally distributed, and binocular accommodative facility, vergence facility, Mid CSF, High CSF, and AULCSF were normally distributed (shown in Figure 3). Total points of the questionnaire in 104 participants ranged from 0 to 16 out of 28 (median = 6, Q1 = 4, Q3 = 8.125). Median of visual acuity (log MAR) was 0.00, the first quartile was −0.08 and the third quartile was 0.00. Parameters of CSF (Low CSF: median = 0.70, Q1 = 0.68, Q3 = 0.73); Mid CSF: *M* = 0.95, *SD* = 0.09; High CSF: *M* = 0.29, *SD* = 0.09; AULCSF: *M* = 1.68, *SD* = 0.14; Cut-off SF: median = 28.30, Q1 = 24.78, Q3 = 31.10), binocular accommodative facility (*M* = 10.29, *SD* = 4.31) and vergence facility (*M* = 12.25, *SD* = 5.50) were detected from participants, respectively.

**FIGURE 4:**
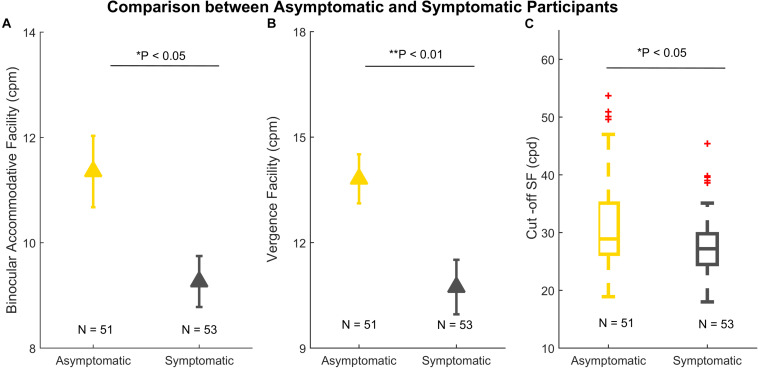
Comparison between asymptomatic (*N* = 51) and symptomatic participants (*N* = 53). **(A)** Binocular accommodative facility. **(B)** Vergence facility. **(C)** A boxplot of Cut-off SF. In panels **(A)** and **(B)**, the error bars denote standard errors. In panel **(C)**, the line within each box is the median. The box spans the interquartile range (Q1 and Q3). The whiskers represent 1.5 × interquartile above the upper quartile (Q3) and below the lower quartile (Q1). The red crosses outside the whiskers are outliers. *p < 0.05; **p < 0.01.

Those with more than one subjective symptom of visual symptoms were categorized as symptomatic of visual fatigue (see Methods). There were significant differences between asymptomatic (*N* = 51) and symptomatic (*N* = 53) participants in binocular accommodative facility [*t(102)* = 2.508, *P* = 0.013], vergence facility [*t(102)* = 2.944, *P* = 0.004], Cut-off SF (*Z* = −2.270, *P* = 0.023), shown in Figure 4. There was no significant difference in AULCSF, Low CSF, Mid CSF, and High CSF.

### Factor Analysis

The scores of the 15 items in the questionnaire showed inter-correlation amongst clusters of items (shown by the Bartlett’s test). For this reason, we performed factor analysis. We found that we could reduce our entire dataset based on four factors (shown in Table 1) because there were four factors which had eigenvalues larger than 1. These four factors are disorientation and difficulty in focusing, discomfort after near work, unclear mind and physical discomfort.

**TABLE 1:** Factor analysis for 15 items in the questionnaire after Varimax with Kaiser Normalization.

	Factor 1 Disorientation and difficulty in focusing	Factor 2 Discomfort after near work	Factor 3 Unclear mind	Factor 4 Physical discomfort
Q1		0.777		
Q2		0.582		
Q3				0.699
Q4	0.600			
Q5	0.781			
Q6	0.526	0.600		
Q7	0.722			
Q8			0.791	
Q9	0.692			
Q10	0.586	0.503		
Q11				0.692
Q12			0.880	
Q13		0.647		
Q14			0.619	
Q15				0.659

*The values are factor loading scores that have been standardized.*

**FIGURE 5:**
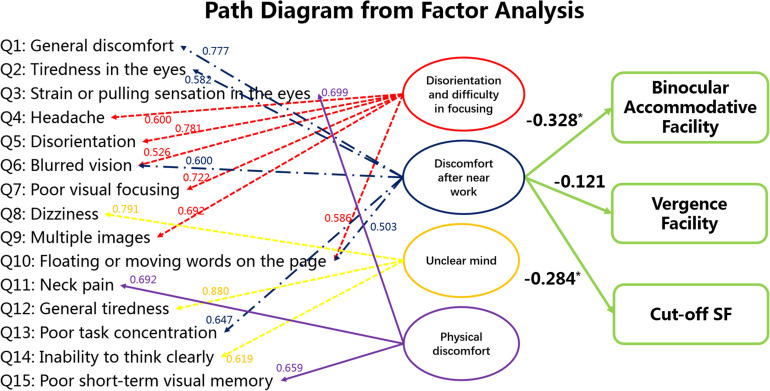
Statistical analysis among subjective symptoms and visual functions (binocular accommodative facility, vergence facility and Cut-off SF). The values between the score of each item in the questionnaire and the latent factors indicate the standardized factor loading scores from the factor analysis. Factor #2 is the focus here because it seems to be strictly concerned with visual fatigue. The values between the latent factor “discomfort after near work” and the visual measures were obtained from bivariate correlation. *p < 0.05.

We believe that factor #2 (discomfort after near work) is most representative of visual fatigue because factor #1 (disorientation and difficulty in focusing) is associated with both physical and visual discomfort, whereas factor #2 is strictly associated with visual discomfort. For this reason, we performed bivariate correlation between the factor “discomfort after near work” and results from clinical visual measurements but not between the other factors and the visual measurements. The results are shown in Figure 5, which indicates that the factor “discomfort after near work” is strongly associated with binocular accommodative facility (Spearman’s *r* = −0.328, *p* < 0.001) and Cut-off SF (Spearman’s *r* = −0.284, *P* = 0.0018), but not with vergence facility (Spearman’s *r* = −0.121, *P* = 0.111).

In addition, multiple linear regression between the factor ‘discomfort after near work’ (Y) and binocular accommodative facility (X_1_) and Cut-off SF (X_2_) was *Y* = −0.215^∗^X_1_ −0.278^∗^X_2_ (*F* = 9.196, *P* < 0.001). The values between the latent factor “discomfort after near work” and the visual measures indicate binocular accommodative facility and Cut-off SF are sensitive measurements that may also inform about visual fatigue.

### Relationships Among the Clinical Visual Measurements

Binocular accommodative facility was correlated positively with vergence facility (Pearson’s *r* = 0.249, *P* = 0.011).

Correlation coefficients between binocular accommodative facility/vergence facility and contrast sensitivity are listed in Table 2. Binocular accommodative facility was moderately correlated with High CSF (Pearson’s *r* = 0.267, *P* = 0.006), but not so with Low CSF (Spearman’s *r* = 0.198, *P* = 0.044) and Mid CSF (Pearson’s *r* = 0.180, *P* = 0.068). Vergence facility was moderately correlated with Low CSF (Spearman’s *r* = −0.271, *P* = 0.005), but not with Mid CSF (Pearson’s *r* = −0.213, *P* = 0.028) and High CSF (Pearson’s *r* = −0.026, *P* = 0.793).

**TABLE 2:** Correlation coefficients (r) between binocular accommodative facility (BAF)/vergence facility (VF) and contrast sensitivity (Low CSF, Mid CSF, High SF) in 104 participants.

	Contrast sensitivity		
	Low CSF	Mid CSF	High CSF
BAF	0.198	0.180	0.267*
VF	−0.271*	–0.213	–0.026

*P < 0.05/6 due to Bonferroni correction.

## Discussion

In our study, we investigated whether there is an association between the scores of the questionnaire about visual fatigue and results from clinical visual measurements. We performed factor analysis to find associations between the exogenous latent factors (ex. visual fatigue) and endogenous scores of the questionnaire. Then we performed a bivariate correlation analysis between the latent factor “discomfort after near work,” which represents visual fatigue, and the results from clinical visual measurements for binocular accommodative facility, vergence facility and contrast sensitivity. Lastly, we assessed whether there is an association between the measures from the clinical visual measurements themselves.

### Relationship Between Visual Fatigue and Visual Clinical Measurements Based on Factor Analysis

Factor analysis showed that visual fatigue, which is represented by the factor “discomfort after near work,” is strongly correlated with the results of binocular accommodative facility and Cut-off SF but not with vergence facility (see Figure 5). This finding is unexpected because both binocular accommodative facility and vergence facility are related to binocular vision. However, as our results from the subsequent section indicate, results between vergence facility and High CSF are not correlated, whereas those between binocular accommodative facility and High CSF are correlated. These findings indicate that binocular accommodative facility captures not only binocular visual function but also the ability of observers to encode visual details (i.e., high spatial frequency content). Hence, our results from factor analysis indicate that visual fatigue is strongly correlated with the ability of the adult observers to encode visual details at the binocular (cut-off SF, binocular accommodative facility) level.

Moreover, the subsequent section (section #2) of our study indicates that binocular accommodative facility is correlated with vergence facility. This finding indicates that binocular accommodative facility, rather than vergence facility, might be a more sensitive index. A high incidence rate of binocular vision dysfunction in Chinese young adults has been reported (Ma et al., 2019). For this reason, it is critical to come up with a sensitive quantitative measurement to evaluate visual fatigue caused by binocular dysfunction.

In our study, all participants had 20/20 or corrected to 20/20 in their visual acuity, but there were some individuals who reported blurred vision. This observation seems to be in line with results from some studies which indicate that the experience of mild symptoms of asthenopia and foggy vision can decrease contrast sensitivity even if the visual acuity remains intact (Quant, 1992; Järvinen and Hyvärinen, 1997). Since high spatial frequency information can appear at a relatively lower contrast, the contrast sensitivity for higher spatial frequency content is much lower in humans. For this reason, a measurement of contrast sensitivity, Cut-off SF, which reveals the best resolution of visual performance, might better capture the ability of human observers to detect and encode the details (Arden, 1978; Marmor, 1986) than the measurement of visual acuity.

These results indicate that symptoms originating from accommodative and binocular dysfunction primarily impact the feeling of eyes and visual performance. Cut-off SF and binocular accommodative facility seem to be robust predictors of visual fatigue.

### Correlations Among Accommodative Facility, Vergence Facility, and Contrast Sensitivity

Our results show that there are moderate correlations between accommodation/vergence and contrast sensitivity. We found that binocular accommodative facility is positively correlated with High CSF (shown in Table 2). Our results are consistent with those from previous studies. To illustrate, Muck (Mucke et al., 2010) reported that dynamic accommodation is related with contrast sensitivity at high spatial frequency. The faster the dynamic response, the more reduced the contrast sensitivity for high but not low spatial frequency (Mucke et al., 2010). To perform well in an accommodative facility test, individuals should have a good contrast sensitivity for high spatial frequency.

We found a strong negative correlation (*p* < 0.01) between vergence facility and Low CSF. This indicates that when Low CSF is lower, vergence facility is better. Vergence causes a rapid shift of the retina image, which causes one to neglect high spatial frequency information (Campbell and Wurtz, 1978; Burr and Ross, 1982). Suppression of low spatial frequency content takes place during convergence eye movement (i.e., vergence dynamics) (Mucke et al., 2013). This recent study agrees to what we report here, namely no correlation between vergence facility and High CSF, and the negative correlation between vergence facility and Low CSF (shown in Table 2).

Moreover, according to our results, symptomatic participants had worse binocular accommodative facility and vergence facility than asymptomatic ones. There are many studies that show similar findings to our results. For example, Hennessey et al. (1984); Levine et al. (1985) and Momeni-Moghaddam et al. (2014) found that accommodative facility and vergence facility are primary functions for accommodation and vergence. They also found that these two functions have a strong relationship with symptoms of visual fatigue (Garcia-Munoz et al., 2014). In addition, accommodative facility and vergence facility are important for reading (Dusek et al., 2011; CITT-ART Investigator Group et al., 2015). Compared to static accommodative amplitude and fusional range, dynamic function, such as facility, could be more pertinent to reading performance (Buzzelli, 1991; Grisham et al., 2007; Palomo-Alvarez and Puell, 2008; Dusek et al., 2010; Quaid and Simpson, 2013).

Moreover, our study uses the qCSF method (Lesmes et al., 2010), which is quick and reliable (Hou et al., 2010; Lesmes, 2010; Hou et al., 2016). In this study, we show that the correlation between clinical characteristics and areal measure from CSF can be used to explain the relationship between binocular dysfunction and visual performance (contrast sensitivity). This approach can be used not just for assessing visual fatigue but also other visual disorders.

In summary, visual fatigue disrupts the binocular ability of adult observers to encode fine details from their visual environment. Binocular accommodative facility and Cut-off SF were sensitive indexes to detect the influence of visual fatigue.

## Data Availability Statement

The raw data supporting the conclusions of this article will be made available by the authors, without undue reservation.

## Ethics Statement

The studies involving human participants were reviewed and approved by the Ethics Committee of Affiliated eye hospital of Wenzhou Medical University. The patients/participants provided their written informed consent to participate in this study.

## Author Contributions

FZ, FH, and WYW contributed to conception and design of the study and performed the statistical analysis. FZ, JM, PH, and BC organized the database. FZ wrote the first draft of the manuscript. All authors contributed to manuscript revision, read, and approved the submitted version.

## Conflict of Interest

The authors declare that the research was conducted in the absence of any commercial or financial relationships that could be construed as a potential conflict of interest.

## Appendix

**Appendix Table 1:** Visual discomfort questionnaire(Yang and James, 2011). Subjects used a pen to mark the degree of visual symptoms (0 to 4) on paper, 0 for not at all and 4 for extremely likely.

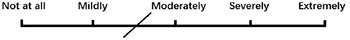

Did you feel physically more uncomfortable in general?
Did your eyes feel more tired?
Did your eyes feel more strain or pulling sensation?
Did your feel your head is fuller or have greater headache?
Did your feel greater disorientation or vertigo?
Did you notice greater blur from the scene you were viewing?
Did you have greater trouble visually focusing on the scene?
Did you feel more severe dizziness?
Did you see multiple images of the scene more?
Did you see the words move, jump, swim or appear to float on the page more?
Did you feel greater neck ache?
Did you feel more tired or sleepy?
Did you have greater difficulty concentrating in the task?
Did you feel like you have greater difficulty thinking clearly?
Did you have greater trouble remembering what you have seen?
